# Prevalence of feco-oral transmitted protozoan infections and associated factors among university students in Ethiopia: a cross-sectional study

**DOI:** 10.1186/s12879-019-4095-z

**Published:** 2019-06-07

**Authors:** Behailu Hawulte Ayele, Ayele Geleto, Desalegn Admassu Ayana, Muhedin Redi

**Affiliations:** 1 School of Public Health, College of Health and Medical Sciences, Haramaya University, Harar, Ethiopia; 2 Department of Medical Laboratory Science, College of Health and Medical Sciences, Haramaya University, Harar, Ethiopia

**Keywords:** *Entamoeba histolytica*/*E. dispar*, *Giardia lamblia*, Feco-oral, Protozoa, Students, University

## Abstract

**Background:**

An estimated 60% of the world’s population is infected with one form of intestinal parasites. Amoebiasis and giardiasis are among the leading intestinal protozoan infections that affected mankind. However, literature that shows the magnitude of the problem among university students in Ethiopia is at scarce. Therefore, this study was aimed at assessing the prevalence of feco-oral transmitted protozoan infections and associated factors among sport festival participant universities in Ethiopia.

**Methods:**

A cross-sectional study design was conducted among 483 randomly selected university sport festival participant students. A self-administered questionnaire was used to collect the data. Stool specimens were examined using direct wet mount and formol-ether concentration techniques. The data were entered into Epi Info version 6.04 and were analyzed using SPSS version 20.0 statistical software. Multivariable logistic regression analysis was done to control the possible confounders and an odds ratio with a 95% confidence interval at *p* < 0.05 was used to identify an association between variables.

**Result:**

The overall prevalence of intestinal protozoan infections was 140(28.9%) with the predominantly higher prevalence of *E. histolytica*/*E. dispar* 95(19.7%). The female respondents were at lower risk of infections compared to their male counterparts (AOR = 0.48, 95% CI: 0.22, 0.97]. Participants with educated father (AOR = 0.62, 95% CI: 0.12, 0.86) and those who received pocket money of > 347 Ethiopian Birr (~ 14 USD) per month (AOR = 0.20, 95% CI: 0.12, 0.74) were at lower risk of infections. However, being married (AOR = 1.42, 95% CI: 1.10, 2.23), rural resident (AOR = 1.82, 95% CI: 1.21, 3.32) and university stay for two or more years (AOR = 2.21, 95%CI: 1.48, 3.87) were more likely to be infected with protozoan infections.

**Conclusion:**

The prevalence of intestinal protozoan infection among students who attend higher educational institutions was very high. Infection prevention strategies should be undertaken at respective universities with special focus to senior students and students from the rural area.

**Electronic supplementary material:**

The online version of this article (10.1186/s12879-019-4095-z) contains supplementary material, which is available to authorized users.

## Background

An estimated 60% of the world population is infected with intestinal parasites including amoebiasis and giardiasis [1, 2]. The incidence of the infections is 50% in developed countries as compared to the incidence rate of 95% in developing countries [2, 3]. The magnitude of intestinal parasitic infections is remarkably higher in tropical and subtropical regions including the sub-Saharan African countries where there are inadequate water supply and basic sanitation [4, 5]. The global burden of intestinal protozoan infections remained unacceptably high despite tremendous achievements in the prevention of communicable diseases. At present, there are approximately 50 million people living with *Entamoeba histolytica* worldwide, while another 3 million people are infected with *Giardia lamblia* [6].

Amoebiasis is an infection caused by *Entamoeba histolytica,* or *E. histolytica, a* protozoan parasite that causes intestinal or extra-intestinal disease [7]. Although it occurs worldwide, the prevalence of the infection is more common in the tropics and sub-tropics regions where there is poor sanitation [4, 8]. Invasive amoebiasis is commonly a disease of young people, and rare among children below five years of age [9]. Amoebiasis is the third common cause of death from parasitic diseases with greater health impact on people who are living in developing countries. Globally, it is estimated approximately 50 million people endure insidious amoebic infection each year, resulting in 40–100 thousand annual deaths [10].

Giardiasis is a protozoan infection and mainly affects the upper parts of the small intestine, usually affects children than adults [7, 11, 12]. *Giardiasis* constitutes a substantial proportion of the 2.5 million annual deaths from diarrheal diseases [13, 14]. In Ethiopia, *giardia* infections that occur during childhood can lead to protein-energy malnutrition and micronutrient deficiency which can be the cause of poor cognitive development and poor educational performance [8].

Like in other developing countries, there is a high prevalence of intestinal protozoan infections in Ethiopia, which could be due to a shortage of clean water, lack of sewage system and poor hygiene [15]. In addition to other common intestinal protozoan parasites, *Giardia lamblia*, *E. histolytica*, and *cryptosporidium* infections are very common in Ethiopia [16].

The magnitude of *E. histolytica/E. dispar* and *G. lamblia* were scarcely studied among university students in Ethiopia*.* However, a study conducted in Wollega University indicated that protozoan diseases remain the serious public health problems due to the low level of environmental sanitation and ignorance of simple health-promoting practices; Students’ hostels are usually unhygienic and unkempt resulting in a high population of flies. The causative agent of intestinal protozoa infections is often transmitted through unhygienic habits including the direct transfer of ova or cysts to mouth, eating with unwashed hands, consuming contaminated food, and poor sanitary conditions [17].

Several studies were conducted in different parts of the world to assess the prevalence of intestinal parasitic infections [12, 17–20]. However, the majority of these studies were focused on preschool children and school children while there is a scarcity of evidence about the magnitude of intestinal protozoa infections among university students. Therefore, this study aimed at assessing the prevalence of feco-oral transmitted protozoan infections, specifically the prevalence and determinants of *E. histolytica/E. dispar* and *G. lamblia,* and associated factors among inter-university annual sports festival participant students. The finding of the study will be used by all public universities found in Ethiopian for planning infection control among universities.

## Methods

### Study design and setting

The cross-sectional study design was conducted among randomly selected higher education students who are attending the annual inter-university sport festival at Haramaya University in 2014. Haramaya University is one of Ethiopia’s eldest universities and its recognized nationally and internationally for excellence in teaching-learning, research and community engagement. It is located 550 km far away from the capital, Addis Ababa. The Ethiopian inter-university sport competition is an annually conducted festival where students from all public universities in the country come together for sport competitions at a selected hosting university. Haramaya University hosted the 7th Public Universities’ sport festival which was conducted from 8th -23rd of February, 2014 [21].

### Sample size and sampling technique

The sample size was determined by a single population proportion formula, [n = (Z α/2)^2^ p (1-p) / d2] [22], by considering the following assumptions. In order to calculate the sample size the prevalence of intestinal protozoan infections 19.9% which is the finding of a study conducted among university students in Wollega University [17], 95% confidence level, 5% margin of error (d = 0.05), and a design effect of 2 were used.

Accordingly, 488 students who are attending higher educational institutions were randomly selected and were included in the study. Multistage sampling technique was employed to identify the universities, colleges, the departments and the study years of the students. The sample was proportionally allocated for all respective universities based on the number of students coming from each university by considering their study year, the colleges and the departments of the student. Finally, the study participants were identified from the list of students by using a simple random sampling method.

### Data collection

The data were collected by using a pre-tested, structured and self-administered questionnaire. The questionnaire was developed by reviewing different literature (see Additional file 1). The questionnaire was developed in the English language after completing a comprehensive literature review. Participants were instructed to complete the questionnaire in a separate room of the Haramaya University higher health center (HUHC) and the stool samples were collected from every participant. The completed questionnaires were recollected and were checked for completeness by an assigned health worker who is working in the health center. For stool investigation, four laboratory technology professionals were recruited and received relevant training on the standard operating procedures and the study objectives.

Approximately 2 g of fresh stool sample was collected using a small and labeled plastic container. The stool samples were then examined at the laboratory of HUHC by using direct wet mount and formol-ether concentration techniques [17, 23]. The direct wet mount technique was chosen because it is relatively cheap, simple, and reliable. In addition, the time required for the procedure was relatively short and requires no sophisticated equipment’s. The wet preparation by using normal saline and iodine solution was simultaneously performed to identify the presence of trophozoite and/or cyst forms.

### Data processing and analysis

The data were entered into Epi-info Version 6.04 and exported to SPSS version 20 statistical packages for analysis. Frequency tables, proportions, a means, and standard deviations were used to describe the variables. An odds ratio was used to determine the presence of an association between explanatory variables and intestinal protozoan infections among the students. The degree of association between dependent and independent variables were assessed using an odds ratio with a 95% confidence interval and *p*-value < 0.05 was taken as a cutoff point to determine the presence of a statistically significant association. For adjustment, variables with *p*-value < 0.2 in bivariate analysis were taken to multivariate analysis. Multivariate logistic regression analysis was employed to measure the relative importance and to control for potential confounders that might affect the effect size and direction of the association between variable.

### Variables

The prevalence of intestinal protozoan infections (represented feco-orally transmitted pathogenic intestinal protozoal infections), is the dependent variable for this study. The independent variables of the study include socio-demographic variables, place of growth, parental educational status, years of study, student’s pocket money, and field of study attended by the students.

### Data quality management

The questionnaire was pre-tested and the feedbacks were used to make modifications to the questionnaires. Members of field staff (the data collectors and two supervisors; one microbiologist to supervise laboratory investigation) were selected based on their experiences of data collection. Before the commencement of the data collection, the data collectors and the supervisors were trained over two days on the objectives of the study, the methods of data collection, and the study tools. Field practice (pre-test) was undertaken to check the applicability of the questionnaire on 5% of the sample size among Haramaya University students who did not participate in the study. The data were checked for its completeness each day by the supervisors and the principal investigators. Laboratory procedures were conducted following the standard operating procedures.

## Results

### Socio-demographic characteristics of the respondents

**Fig. 1:**
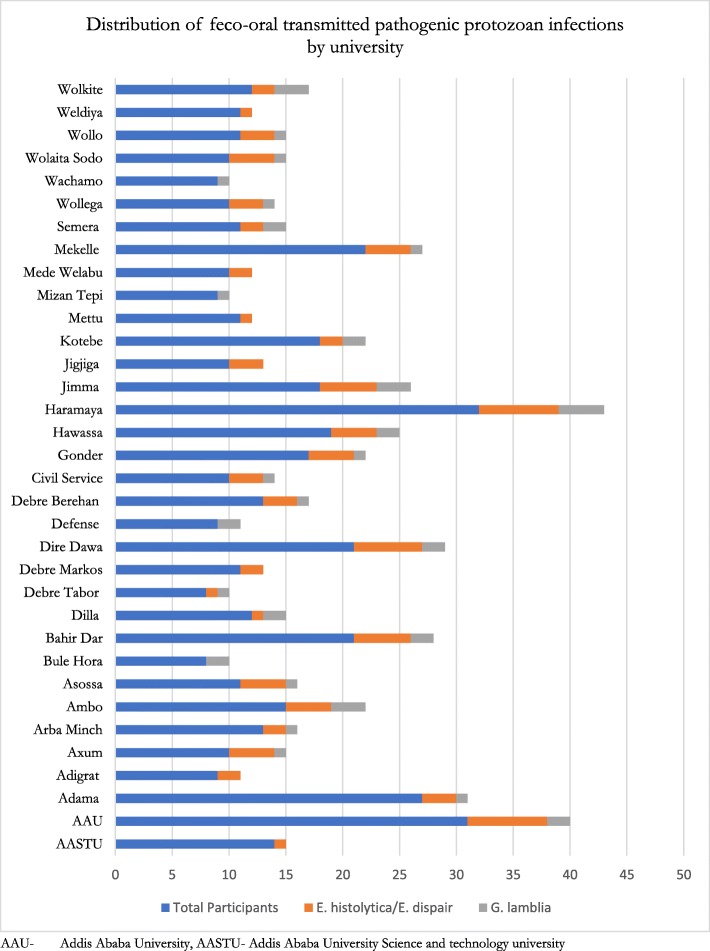
Distributions feco-oral transmitted pathogenic Protozoal infections by university, Ethiopia 2014

From the total of 488 students participated in the study, 5 were excluded due to incomplete responses (making the response rate of 98.9%). The majority of the respondents, 77.8% were male with the mean age of 22 (ranged from 16 to 35) years. Almost all of the respondents, 94.4% were single whereas 65% of the respondents came from the rural areas. Regarding parental education, nearly half of the respondents (49.5%) have an uneducated father while the fathers of the remaining half had attended formal education (Table 1). The average received pocket money was 347 Ethiopian Birr (ETB) (~ 14 USD) per month. One hundred and seven (22.2%) of the respondents were year I (freshman) students and 8.9% of participants were from Haramaya University (Fig. 1).

**Table 1:** Socio-demographic characteristics of the respondents (*n* = 483), Haramaya University, Ethiopia, 2014

Variables	Frequency	Percent
Sex		
Male	375	77.7
Female	108	22.3
Ethnicity		
Amhara	188	38.9
Oromo	138	28.6
Tigrway	69	14.3
Gurage	18	3.7
Others^a^	70	14.5
Religion		
Orthodox	294	61.0
Protestant	100	20.7
Muslim	66	13.6
Catholic	15	3.0
Others^b^	8	1.7
Marital status		
Single	427	88.4
Married	27	5.6
In relationship	29	6.0
Residence		
Rural	314	65.1
Urban	169	34.9
Paternal educational status		
Uneducated	238	49.5
Educated	244	50.5
Year of study		
First year(freshmen)	107	22.2
Second year and above(seniors)	376	77.8
Monthly received pocket money (ETB)		
≤ 347	185	38.3
> 347	298	61.7
Source of pocket money		
Family	395	81.8
Other sources^c^	88	18.2
Field of study		
Natural science	398	82.4
Social science	85	17.6

^a^Sidama, Somali, Wolaita, Kembata, Harari, Hadeya, Silite, Afara, Sinasha

^b^Wakefeta (Indigenous Beliefs) [5], Jehovas witness [3]

^c^Relatives, coast sharing payment (for non-café students), Pay (for students who were engaged in any income generating activities, charity organization (for students supported by charity organization)

### Prevalence of intestinal protozoan infections

Based on the analysis of one formalin-fixed stool sample per a student, 140 (28.9%) of the respondents harbored at least one infection with an intestinal protozoa species. The prevalence of *E. histolytica*/*E. dispar* among the respondents was 95(19.7%); 71(14.7%) cyst stage and 24(5%) trophozoite stage. Similarly, 45(9.3%) of the students were positive for *G. lamblia;* 20(4.1%) cyst and 25 (5.2%) its trophozoite (Table 2). The magnitudes of infections were higher among males (19.5%) than females (9.4%).

**Table 2:** Result of stool examination for intestinal protozoa parasitic infections of participants (*n* = 483), Haramaya University, Ethiopia, 2014

Intestinal prtozoan infections	Infected, n (%)	Not infected, n (%)
*E. histolytica* */E. dispair*	95(19.7)	338(80.3)
Cyst	71(14.7)	412(85.3)
Trophozoite	24(5.0)	459(95.0)
*G. lamblia*	45(9.3)	438(90.7)
Cyst	20(4.1)	463(95.9)
Trophozoite	25(5.1)	458(94.9)
Overall positive for Protozoa infections	140(28.9)	343(70.1)
Cyst	91(18.8)	392(81.2)
Trophozoite	49(10.1)	434(89.9)

### *Factors associated with* protozoa infections

The multivariate logistic regression analysis showed that gender, residence, paternal education, the study year, monthly income and marital status were significantly associated with protozoa infections. Female participants were less likely to be infected with protozoa infections compared to males (AOR = 0.48, 95% CI: 0.22, 0.97). Married students were more likely to have acquired protozoa infections than those who never married (AOR = 1.42, 95% CI: 1.10, 2.23). Students who came from the rural areas were about 1.82 times more likely to be infected with protozoa infections as compared to those who came from the urban (AOR = 1.82, 95% CI: 1.21, 3.32). Participants who attended year II and above were two times more likely to be infected as compared to year I (freshman) (AOR = 2.21, 95% CI: 1.48, 3.87). Students who have an educated father were less likely to have infections as compared to those who have uneducated fathers (AOR = 0.62, 95% CI: 0.12, 0.86). Study participants whose monthly received pocket money was > 347 ETB (~ 14 USD) were less likely to be infected with protozoa infections (AOR = 0.20, 95%CI:0.12, 0.74) (Table 3).

**Table 3:** Factors associated with protozoa infections among respondents, Haramaya University, Ethiopia 2014

Variables	Protozoa infections		COR (95%CI)	AOR (95%CI)
	Positive	Negative		
Sex				
Male	98	277	1	1
Female	42	66	0.55(0.12,0.86)	0.48(0.22,0.97) *
Ethnicity				
Amhara	52	136	1	1
Oromo	37	101	1.04(0.42,3.29)	1.21(0.34,3.17)
Tigrway	18	51	1.08(0.30,2.65)	1.06(0.41,2.43)
Gurage	9	9	0.38(0.14,1.81)	0.29(0.17,1.62)
Others	24	46	0.73(0.22,1.86)	0.64(0.24,1.62)
Religion				
Orthodox	96	198	1	
Protestant	22	78	1.71(0.60,3.47)	1.56(0.48,3.18)
Muslim	16	50	1.51(1.12,3.38)	1.45(0.93,2.87)
Catholic	4	11	1.33(0.78,4.81)	1.42(0.65,4.72)
Others	2	6	1.48(0.10,2.96)	1.39(0.45,3.24)
Marital status				
Single	134	322	1	1
Married	6	21	1.45(1.11,3.54)	1.42(1.10,2.23) *
Residence				
Urban	64	105	1	
Rural	76	238	1.90(1.24,3.45)	1.82(1.21,3.32) *
Paternal educational status				
Uneducated	62	176	1	1
Educated	78	166	0.75(0.32,0.94)	0.62(0.12,0.86) *
Year of study				
First year	48	59	1	
Second year and above	92	284	2.51(1.24,4.23)	2.21(1.48,3.87) *
Monthly received pocket money (ETB)				
≤ 347	22	163	1	
> 347	118	180	0.20(0.10,0.76)	0.20(0.12,0.74) *
Source of pocket money				
Family	121	274	1	
Other sources	19	69	1.60(1.24,2.81)	1.52(0.18,2.64)
Field of study				
Natural science	113	285	1	
Social science	27	58	0.85(0.63,1.79)	

*-indicates P-Value less than 0.05

## Discussion

This study revealed that 140 (28.9%) of the students were infected with either *E. histolytica*/*E. dispar* or *G. lamblia*. Being a female, having an educated father and earning more than 347 ETB (~USD 14) were found to be protective against protozoan infections. However, being a single, coming from a rural area and being a senior student were found to be risk factors for protozoan infections.

The magnitude of *E. histolytica*/*E. dispar* or *G. lamblia* in the current study was higher compared to a previous study conducted in Ethiopia; Wollega University, 19.9% [17], Benishangul-Gumuz region, 26.6% [24], Nigeria; Michael Okpara University, 9.3% [25] and Akanu Ibiam Federal Polytechnic college, 21.2% [26], and Tanzania, 20.5% [27]. The reason for the high magnitude in this study may be attributed to poor personal hygiene due to a shortage of sanitary water supply in most parts of Ethiopia including universities [28, 29]. Moreover, poor food handling practices, consumption of contaminated food, poor sanitary conditions at the students’ cafeteria [17, 30], and poor hand washing practice of the students [31, 32] may have contributed to the higher prevalence.

The findings of our study indicated that *E. histolytica/E. dispar* was more prevalent 95(19.7) than *G lamblia* which is 40 (9.3%) among the students. Consistent with our study finding, studies conducted in the Gamo Gofa zone of southern Ethiopia [20] and in Saudi Arabia [33] revealed that more study participants were infected with *E. histolytica/E. dispar* than *G. lamblia*. This might be explained by poor personal hygiene among cafeteria workers and serving inadequately washed vegetables [30]. It may also be attributed to the nature of transmission of the infections and the infectiousness period [7].

In the current study, a higher magnitude of feco-orally transmitted protozoan infections was observed among male students as compared to their female counterparts. Similar findings that support our results were reported from the study conducted at Wollega University [17], where 12.6% male versus 7.4% female students were infected. The higher magnitude of infections among male is likely to be a reflection of different levels of hygienic behaviour between the genders [31]. Inversely, the inconsistent finding was reported from the Ebonyi state, Nigeria, where 36.0% of female versus 18.3% male were infected [25] and Tanzania, where 58.1% of females versus 42.3% of males [27]. The higher prevalence of infections among males than females may be attributed to that males usually consumes unwashed fruits and vegetables or uncooked salads which may be contaminated with the protozoan cysts [34, 35].

The current study revealed that students who come from a rural area were at an increased a risk for the protozoan infections. Similar findings were reported from the study conducted in Wollega university which identified students from rural areas acquire more infection than students from urban areas [17]. A consistent finding was also reported from the study conducted in Nigeria where rural dwellers were more prone to protozoan infections [19]. This might be explained by the difference in the lifestyles of urban and rural residents. Moreover, students from rural areas might be engaged in farming activities and usually use water from unprotected wells or rivers which are more likely to be contaminated [3, 36].

### Limitations

This study may not indicate the causal relationship among variables because we employed a cross-sectional study design, which cannot establish a casual effect. The study included information about students who participated in the sport festival and may not be generalized to the general university students. The paucity of literature on the feco-oral transmitted protozoan infections among university students to compare our findings was another important limitation.

## Conclusion

Prevalence of intestinal protozoan infection among students was very high. This high prevalence indicated that much work related to sanitation and hygiene has to be done to prevent intestinal protozoa infections and improve the health of the students. Universities should strengthen health promotions and educations on personal and environmental hygiene in order to improve sanitation for university students. Since protozoan infection can be acquired through feco-oral contact with contaminated food and drink, sanitation of students’ cafeteria, food handlers, the foodstuff, and the water should be taken into consideration to reduce food-and water-borne protozoan infections.

## Additional file

Additional file 1: The questionnaire. The questionnaire used to assess Prevalence of feco-oral transmitted protozoan infections and associated factors among university students in Ethiopia: A Cross-Sectional Study. (DOCX 27 kb)

## Abbreviations

AAU: Addis Ababa University
AOR: Adjusted odds ratio
CI: Confidence interval
COR: Crude odds ratio
ETB: Ethiopian birr
HU: Haramaya University
HUHC: Haramaya University Health Center
IHRERC: Institutional Health Research Ethics Review Committee
OR: Odds ratio
SD: Standard deviation
SPSS: Statistical package for social sciences
USD: United State dollar
WHO: World health organization

## References

[1] WHO (1987). Prevention and Control of intestinal parasitic infections. Report of a WHO expert committee. World Health Organ Tech Rep Ser.

[2] Chacon-Cruz E (2003). Intestinal Protozoal Diseases. eMedicine J.

[3] Romano Ngui SI, Chuen CS, Mahmud R, Lim YAL (2011). Prevalence and risk factors of intestinal parasitism in rural and remote West Malaysia. PLoS Negl Trop Dis.

[4] Haque R (2007). Human intestinal parasites. J Health Popul Nutr.

[5] Fletcher SM, Stark D, Ellis J (2011). Prevalence of gastrointestinal pathogens in sub-Saharan Africa: systematic review and meta-analysis. J Public Health in Africa.

[6] Samuel LSJ, Reed SL (2001). Microbes and microbial toxins: paradigms for MicrobialMucosal interactions. Entamoeba histolytica: parasite-host interactions. Am J Physiol Gastrointest Liver Physiol.

[7] Wright S (2005). Amoebiasis and giardiasis. J Med.

[8] Alemayehu M. Lecture notes on communicable disease control Addis Abeba the Carter Center, Ethiopia Ministry of Health, and Ethiopia ministry of education; 2004.

[9] VIC H (2015). Communicable disease epidemiology and surveillance.

[10] Dhanabal Jeevitha, Selvadoss Pradeep Pushparaj, Muthuswamy Kanchana (2014). Comparative Study of the Prevalence of Intestinal Parasites in Low Socioeconomic Areas from South Chennai, India. Journal of Parasitology Research.

[11] Choy SH, Al-Mekhlafi HM, Mahdy MAK, Nasr NN, Sulaiman M, Lim YAL (2014). Prevalence and associated risk factors of Giardia infection among indigenous communities in rural Malaysia. Sci Rep.

[12] Anim-Baidoo I, Narh CA, Oddei D, Brown CA, Enweronu-Laryea C, Bandoh B (2016). Giardia lamblia infections in children in Ghana. Pan Afr Med J.

[13] Pires SM, Fischer-Walker CL, Lanata CF, Devleesschauwer B, Hall AJ, Kirk MD (2015). Aetiology-specific estimates of the global and regional incidence and mortality of Diarrhoeal diseases commonly transmitted through food. PLoS One.

[14] Younas M, Shah S, Talaat A (2008). Frequency of Giardia lamblia infection in children with recurrent abdominal pain. JPMA J Pak Med Assoc.

[15] Haileeyesus Adamu BP (2009). Intestinal protozoan infections among HIV positive persons with and without antiretroviral treatment (ART) in selected ART centers in Adama, Afar and DireDawa, Ethiopia. Ethiop J Health Dev.

[16] Yemane B (2005). Epidemiology of Health and Disease in Ethiopia.

[17] Asegid A, Desalegn A (2015). Prevalence of intestinal protozoan among students visiting Wollega University students’ clinic. Adv Med Biol Sci Res.

[18] Aschalew G, Belay A, Bethel N, Betrearon S, Atnad Y, Meseret A, et al. Prevalence of intestinal parasitic infections and risk factors among schoolchildren at the University of Gondar Community School, Northwest Ethiopia: a cross-sectional study. BMC Public Health. 2013;13(304). 10.1186/1471-2458-13-304. 10.1186/1471-2458-13-304 PMC3621079 23560704

[19] Bernard OA (2014). INOaSEY. Prevalence of pathogenic protozoa infection in humans and their associated risk factors in Benue state, Nigeria. Int J Public Health Epidemiol.

[20] Teklu Wegayehu, Tsegaye Tsalla, Belete Seifu, and Takele Teklu. Prevalence of intestinal parasitic infections among highland and lowland dwellers in Gamo area, South Ethiopia. BMC Public Health. 2013;13(151). 10.1186/1471-2458-13-151. 10.1186/1471-2458-13-151 PMC3584849 23419037

[21] HU. Haramaya University: Wikipedia, the free encyclopedia; 2014 Available from: https://en.wikipedia.org/wiki/Haramaya_University.

[22] Dean AG, Sullivan KM, Soe MM. OpenEpi: Open Source Epidemiologic Statistics for Public Health, Emory University, Rollins School of Public Health.Version. 3.01, updated 2013/04/06. www.OpenEpi.com. Accessed 18 May 2019.

[23] Cheesbrough M, Technology TH (2009). District laboratory practice in tropical countries.

[24] Tigabu EPB, Endeshaw T (2010). Prevalence of giardiasis and cryptosporidiosis among children in relation to water sources in selected village of Pawi special district in BenishangulGumuz region, northwestern Ethiopia. Ethiop J Health Dev.

[25] Ngele KK (2012). The prevalence of intestinal protozoan parasites among the undergraduate students of Akanu Ibiam Federal Polytechnic, Unwana, Ebonyi state Nigeria. Int J Sci Nat.

[26] CCOaNB O (2013). Intestinal parasites among undergraduate students of Michael Okpara University of Agriculture, Umudike Abia state, Nigeria. World Appl Sci J.

[27] Mazigo HD, Ambrose EE, Zinga M, Bahemana E, Mnyone LL, Kweka EJ, Heukelbach J. Prevalence of intestinal parasitic infections among patients attending Bugando medical Centre in Mwanza, North-Western Tanzania: a retrospective study. Tanzan J Health Res. 2010;12(3).

[28] Seifu A, Amy S, Manyahleshal A. Water supply and sanitation in Amhara Region. In: Learning and Communication Research Report, Bahir Dar, Ethiopia; 2012.

[29] Ethiopia WA (2010). Regional water supply and sanitation coverage in Ethiopia: according to 2001 EFY reports.

[30] Aklilu Addis, Kahase Daniel, Dessalegn Mekonnen, Tarekegn Negatu, Gebremichael Saba, Zenebe Seyfe, Desta Kassu, Mulugeta Gebru, Mamuye Yeshiwodim, Mama Mohammedaman (2015). Prevalence of intestinal parasites, salmonella and shigella among apparently health food handlers of Addis Ababa University student’s cafeteria, Addis Ababa, Ethiopia. BMC Research Notes.

[31] Simon Mariwah KH, Kasim A (2012). The impact of gender and physical environment on the handwashing behaviour of university students in Ghana. Trop Med Int Health.

[32] Marufa S, Rashidul AM, Abdur RS, Sarder MH. Hand hygiene knowledge and practice among university students: evidence from private universities of Bangladesh. Dove Press. 2015;2016(9):13–20. 10.2147/RMHP.S98311. 10.2147/RMHP.S98311 PMC4758791 26929673

[33] Zaglool DAKY, Gazzaz ZJ, Dhafar KO, Shaker HA (2011). Prevalence of intestinal parasites among patients of Al-Noor specialist hospital, Makkah, Saudi Arabia. Oman Med J.

[34] Al-Rifaai JM, Al Haddad AM, Qasem JA (2018). Personal hygiene among college students in Kuwait: a health promotion perspective. J Educ Health Promot.

[35] Prah J, Abdulai M, Lasim O, Ampofo-Asiama A (2018). Assessment of hygiene Practices.

[36] Mainous AG, Kohrs FP (1995). A comparison of health status between rural and urban adults. J Community Health.

