# Supplementary material for: Prevalence of feco-oral transmitted protozoan infections and associated factors among university students in Ethiopia: a cross-sectional study
Source: BMC Infect Dis. 2019 Jun 7;19:499. doi: 10.1186/s12879-019-4095-z (PMC6555915; doi:10.1186/s12879-019-4095-z)
Supplement: Supplementary file 1 — The questionnaire. The questionnaire used to assess Prevalence of feco-oral transmitted protozoan infections and associated factors among university students in Ethiopia: A Cross-Sectional Study. (DOCX 27 kb) [file 12879_2019_4095_MOESM1_ESM.docx]

## **Questionnaire (English Version)**

University code __________________________

Interview Code _______________________ Date: _______/_______/_____________(dd/mm/yyyy)

Name and signature of data collector _______________________________ __________________

Name and signature of data collector supervisor __________________________ _____________

**Information Sheet and voluntary informed consent for the university’s administration**

***Preface:*** My Name is ________________________________. I am working as a data collector for the study being conducted by Mr. Behailu Hawulte, Ayele Geleto, Desalegn Admassu, and Muhedin Redi who are currently working at Haramaya University, College of Health and Medical Sciences.

***Title of the study:*** Prevalence of feco-oral transmitted protozoan infections and associated factors among university students in Ethiopia

***Purpose/aim of the study:*** The aim of the study is to assess the prevalence feco-oral transmitted pathogenic protozoan infections and associated factors among university students. This will contribute to have necessary information for decision in planning and programming appropriate intervention to prevent/reduce occurrences of the problem.

***Procedure and duration:*** Randomly selected students will be given questionnaires to be filled to get pertinent data that is helpful for the study. After completion of the questionnaires, students will be requested approximately 2 g of fresh stool sample using a small and labeled plastic container for stool examination.

***Risk and benefits:*** The risk of participating in this study is very minimal for students and your universities, only taking few minutes (30 minutes) for interview and stool examination. There would not be any direct payment for participating in this study. However, in case students are found to be infected with feco-oral transmitted protozoan infection, they will get appropriate treatment free of charge. In addition, the findings from this study may reveal important information for concerned bodies.

***Confidentiality:*** The information obtained from students will be confidential. There will be no information that will identify your organization and students in particular. The findings of the study will be general for the study population and will not reflect anything particular of individual person or your organization. The questionnaire will be coded to exclude showing students and university names. No reference will be made in oral or written reports that could link participants/ organization to the study.

***Rights:*** Participation for this study is fully voluntary. Students have the right to declare to participate or not in this study. If students decide not to participate, they have the right to withdraw from the study at any time, this will not able students for any loss of benefit which they otherwise are entitled. Students do not have to answer any question that they do not want to answer.

***Contact address:*** If there is any question or enquiry any time about the study or the procedure, please contact: at (+251)-0912186283, Behailu Hawulte (Principal Investigator), as well as Institutional Health Research Ethics Review Committee (IHRERC) of Haramaya University, College of health sciences at office phone (+251)-025-466-07-08 or P. O. Box 235, Harar, Ethiopia.

***Declaration of institutional Informed Voluntary Consent:*** I have read the institutional information sheet and voluntary informed consent. I have clearly understood the purpose of the research, the procedures, the risk and benefits, issues of confidentiality, the rights of participating, and contact address for any enquiry. I have been given the opportunity to ask questions for things that may have been unclear. I was informed that I have the right to stop study at any time if any issues uncomfortable raised by the clients or other concerned body. Therefore, on behalf of my institution, I declared my voluntary consent to permit the study with my initials (signature) as indicated below:

Signature of the university representative: ________________________ _______________

Signature of data collector: _____________________________ _______________

Date: ________/_________/_____________

## **Questionnaire (English Version)**

University code __________________________

Interview Code _______________________ Date:_______/_______/_____________(dd/mm/yyyy)

Name and signature of data collector _______________________________ __________________

Name and signature of data collection supervisor __________________________ _____________

**Information Sheet and voluntary informed consent for the students**

***Preface:*** My Name is ________________________________. I am working as a data collector for the study being conducted by Mr. Behailu Hawulte, Ayele Geleto , Desalegn Admassu, and Muhedin Redi who are currently working at Haramaya University, College of Health and Medical Sciences.

***Title of the study:*** Prevalence of feco-oral transmitted protozoan infections and associated factors among university students in Ethiopia

***Purpose/aim of the study:*** The aim of the study is to assess the prevalence feco-oral transmitted pathogenic protozoan infections and associated factors among university students. This will contribute to have necessary information for decision in planning and programming appropriate intervention to prevent/reduce occurrences of the problem.

***Procedure and duration:*** I will give you questionnaires prepared to collect pertinent information for the study and you will kindly be requested to fill the questionnaire. After completion of the questionnaires, you will be requested approximately 2 g of fresh stool sample using a small and labeled plastic container for stool examination.

***Risk and benefits:*** The risk of participating in this study is very minimal, only taking few minutes (30 minutes) for interview and stool examination. There would not be any direct payment for participating in this study. However, in case you are infected with feco-oral transmitted protozoan infection, you will get appropriate treatment free of charge. More importantly, the findings from this study will be used to intervene for identified problems.

***Confidentiality:*** The information you provide will be confidential. There will be no information that will identify you in particular. The findings of the study will be general for the study population and will not reflect anything particular to individual person. The questionnaire will be coded to exclude showing your name. No reference will be made in oral or written reports that could link participants/ organization to the study.

***Rights:*** Participation for this study is fully voluntary. You have the right to declare to participate or not in this study. If you decide not to participate, you have the right to withdraw from the study at any time, this will not able you for any loss of benefit which you otherwise are entitled. you do not have to answer any question that you do not want to answer.

***Contact address:*** If there is any question or enquiry any time about the study or the procedure, please contact: at (+251)-0912186283, Behailu Hawulte (Principal Investigator), as well as Institutional Health Research Ethics Review Committee (IHRERC) of Haramaya University, College of health sciences at office phone (+251)-025-466-07-08 or P. O. Box 235, Harar, Ethiopia.

***Declaration of Informed Voluntary Consent:*** I have read the institutional information sheet and voluntary informed consent. I have clearly understood the purpose of the study, the procedures, the risk and benefits, issues of confidentiality, the rights of participating, and contact address for any enquiry. I have been given the opportunity to ask questions for things that may have been unclear. I was informed that I have the right to stop at any time if any issues uncomfortable. Therefore, I declared my voluntary consent to participate on the study with my initials (signature) as indicated below:

Signature of the participant: ________________________ _______________

Signature of data collector: _____________________________ _______________

Date: ________/_________/_____________

## Questionnaires

**A. Socio demographic characteristics**

| **Code** | **Variable** | **Response** | **Skip** |
| --- | --- | --- | --- |
| Q001 | Sex | Male ………………………….1 Female…………………………2 |  |
| Q002 | Age | __________ in years |  |
| Q003 | University | ____________ |  |
| Q004 | College | _____________ |  |
| Q005 | Department | ______________ |  |
| Q006 | Year of Study | ____________ |  |
| Q007 | Field of study |  |  |
| Q008 | Recent semester Cumulative Grade Point Average (CGPA) | _____________ |  |
| Q009 | Level of parental education | Uneducated …………………………1  Primary education (1-8 grade) ………2  High school (9-12) ………………… 3 College /university ………………… 4 |  |
| Q010 | Marital status | Married …………………………...1 Divorced………………………….2 Widowed…………………………3  Single or never married………….4 |  |
| Q011 | Religion | Muslim ……………………………1 Orthodox……………….…………2 Protestant …………………………3 Catholic ………………...…………4 Others(specify)______________...5 |  |
| Q012 | Ethnicity | Oromo…………………………1  Amhara ……………………….…2  Tigray …………………………….3  Gurage…………………………….4  Others (specify)_________________........9 |  |
| Q013 | Residence | Urban …………………………1 Rural ………………….………2 |  |
| Q014 | Monthly received pocket money (ETB) | ___________________ETB |  |
| Q015 | Source of pocket money | Family………………………1  Relatives ……………………2  Coast sharing payment……………………3  Pay………………………….4  Charity organization………….5  Other sources (specify) ____________________________...........9 |  |

**B. Stool examination result report**

Date____________

Time: _____: _____

Interview code ______________________ Age _________ Sex___________

| Code | Request | Result | Remark |
| --- | --- | --- | --- |
| Q101 | Stool Examination Result |  |  |
| Tick on “YES” or “NO”, based on the protozoal agents you have detected through stool examination | | | |
| Q102 | Cyst of *E. histolytica/E. despair* | Yes …………………………1  No …………………………2 |  |
| Q103 | Trophozoite of *E. histolytica/E. despair* | Yes …………………………1  No …………………………2 |  |
| Q104 | Cyst *G. lamblia* | Yes …………………………1  No …………………………2 |  |
| Q105 | Trophozoite of *G. lamblia* | Yes …………………………1  No …………………………2 |  |
| Q106 | Cryptosporidium spp | Yes …………………………1  No …………………………2 |  |
| Q107 | Others (specify)  1.______________________________________________  2. ______________________________________________  3. ______________________________________________  4. ______________________________________________ | |  |

Sign of Lab Tech. ____________________ Date ______________________
